# Exploring the mechanisms underpinning sweating: the development of a specialized ventilated capsule for use with intradermal microdialysis

**DOI:** 10.14814/phy2.12738

**Published:** 2016-03-31

**Authors:** Robert D. Meade, Jeffrey C. Louie, Martin P. Poirier, Ryan McGinn, Naoto Fujii, Glen P. Kenny

**Affiliations:** ^1^ Human and Environmental Physiology Research UnitFaculty of Health SciencesUniversity of OttawaOttawaOntarioCanada

**Keywords:** Heat loss, heat stress, sweat rate, thermoregulation

## Abstract

Many studies have aimed to identify the controllers of sweating using ventilated capsules with intradermal microdialysis. It is unclear, however, if the surface area covered by the capsule influences the observed response as a result of differences in the number of sweat glands affected by the infused pharmacological agent relative to the total glands captured by the capsule. We evaluated the area of skin perfused with agents delivered via microdialysis. Thereafter, we developed a specialized sweat capsule (1.1 cm^2^) and compared the sweating response with a classic capsule (2.8 cm^2^). In *Protocol 1* (*n* = 6), methacholine was delivered to forearm skin in a dose‐dependent manner (1–2000 mmol L^−1^). The area of activated sweat glands was assessed via the modified iodine‐paper technique. In *Protocol 2* (*n* = 6), the area of inhibited sweat glands induced by ouabain and atropine was assessed during moderate‐intensity cycling. Marked variability in the affected skin area was observed (0.9 ± 0.4 to 5.2 ± 1.1 cm^2^). In *Protocol 3* (*n* = 6), we compared the attenuation in local sweat rate (LSR) induced by atropine between the new and classic capsule during moderate‐intensity cycling. Atropine attenuated sweating as assessed using the new (control: 0.87 ± 0.23 mg min^−1^ cm^−2^ vs. atropine: 0.54 ± 0.22 mg min^−1^ cm^−2^; *P* < 0.01) and classic (control: 0.85 ± 0.33 mg min^−1^ cm^−2^ vs. atropine: 0.60 ± 0.26 mg min^−1^ cm^−2^; *P* = 0.05) capsule designs. Importantly, responses did not differ between capsule designs (*P* = 0.23). These findings provide critical information regarding the skin surface area perfused by microdialysis and suggest that use of a larger capsule does not alter the mechanistic insight into the sweating response gained when using microdialysis.

## Introduction

The evaporation of sweat from the skin is a critical mechanism governing human thermoregulation, especially during exercise and/or exposure to a hot environment. In fact, when ambient temperature exceeds that of the skin, sweating is the major mechanism by which heat loss occurs. As a consequence, different means of assessing sweat production have been developed with the most common method being the ventilated capsule technique, in which dry gas (typically air or nitrogen) is passed through a capsule affixed to the skin. By evaluating the change in humidity between the effluent and influent air streams using a hygrometer, local sweat rate can then be calculated. Using this technique, major insights in our understanding of the influence of thermal and nonthermal modulators of sweat rate during heat stress have been achieved (Shibasaki and Crandall 2010; Kenny and Jay 2013). However, despite these advancements, the mechanisms and controllers associated with the modulation of the sweating response have, until recently, remained largely unresolved.

An increasing number of studies have employed the ventilated capsule technique in combination with intradermal microdialysis (which allows for the direct delivery of pharmacological agents through a semipermeable membrane inserted into the skin) to investigate the putative transmitters and cotransmitters involved in the regulation of sweating at the level of the end organ – the sweat gland. Agents are chosen to stimulate or inhibit specific receptors and/or enzymes and, based on the sweating response relative to a control site, mechanistic insight may be gathered. To this end, new knowledge related to our understanding of the mechanisms associated with the influence of local temperature (Wingo et al. 2010), age (Smith et al. 2013; Stapleton et al. 2014b), sex (Gagnon et al. 2013), and acclimation status (Lorenzo and Minson 2010), to name a few, has been gleaned. Furthermore, this methodological approach has yielded new insights into the roles of extracellular calcium, nitric oxide, cyclooxygenase, and angiotensin II in the modulation of forearm sweating (Welch et al. 2009; Fujii et al. 2014, 2015d; Metzler‐Wilson et al. 2014; Stapleton et al. 2014a) while also assessing the contribution of other compounds such as ATP and adenosine (McGinn et al. 2014; Fujii et al. 2015a,b).

Despite the important benefits that intradermal microdialysis provides in advancing our understanding of the underlying physiology of sweating, there are some potential limitations to its use that must be addressed. Given that the perfusion area of the pharmacological agents may differ as a function of its diffusion properties and its concentration, the size of the ventilated capsule and therefore the number of sweat glands captured by the capsule may impact the observations. Specifically, the surface area covered by the capsule may influence the sweating response as a result of differences in the number of sweat glands affected by the infused pharmacological agent relative to the total glands captured by the capsule. This is an especially important consideration for the evaluation of the mechanisms underpinning sweating given the variety of different capsule sizes employed between studies (Table 1). Thus, the purpose of this study was to estimate the surface area of skin perfused by intradermal microdialysis using both an agonistic (in which the sweat glands are activated pharmacologically) and inhibitory (in which activated sweat glands during exercise are inhibited) approach. From these data, a specialized sweat capsule was designed for use with intradermal microdialysis. We then sought to compare the local sweating response measured with this newly designed capsule to those observed with use of a classic (i.e., larger) capsule design. We hypothesized that, relative to a classic capsule, this new ventilated capsule would better capture the area of sweat glands perfused via microdialysis during exercise at a fixed rate of metabolic heat production (ensuring a consistent thermal drive for sweating), as evidenced by a comparatively greater attenuation in the measured local sweating response induced by perfusion of atropine.

**Table 1:** Range of reported ventilated sweat capsule surface areas across selected studies (utilizing intradermal microdialysis) to highlight the variety of capsule sizes that have been employed

Study	Sweating stimulus	Capsule surface area
Lorenzo and Minson (2010)	Cholinergic stimulation (acetylcholine)	0.5 cm^2^
Welch et al. (2009)	Exercise (60% VO_2peak_)*	0.567 cm^2^
Metzler‐Wilson et al. (2014)	Cholinergic stimulation (acetylcholine)	0.6 cm^2^
Wingo et al. (2010)	Whole‐body passive heating‡	0.64 cm^2^
Lee and Mack (2006)	Cholinergic stimulation (methacholine)	0.7 cm^2^
Fujii et al. (2015c)	Exercise†	2.8 cm^2^
Wilson et al. (2005)	Whole‐body passive heating‡	2.83 cm^2^
Schlereth et al. (2006)	Cholinergic stimulation (acetylcholine)	3.125 cm^2^
Smith et al. (2013)	Cholinergic stimulation (acetylcholine) and whole‐body passive heating‡	4.46 cm^2^

Sweating stimulus refers to the means by which sweating was induced. Cholinergic stimulation was accomplished by administration of exogenous cholinergic agonists (i.e., acetylcholine or methacholine).

Exercise models were performed *at a fixed percentage of participant's peak oxygen consumption (VO_2peak_) or ^†^at a fixed rate of metabolic heat production.

^‡^Whole‐body passive heating achieved with use of a water perfused suit.

## Methods

### Participants

A total of 11 habitually active (2–4 days per week of structured physical activity, ≥30 min in duration) young adults (seven males, four females) participated in at least one of the three experimental protocols performed in the current study (see below for a description of each protocol). All participants were free of cardiovascular, metabolic, and/or respiratory disease. Furthermore, participants had no history of autonomic or skin disorders, were nonsmoking, and were not taking any prescription or over‐the‐counter medications at the time of the study. All female participants completed the study during the early follicular phase (within 6 days of starting menstruation) or placebo phase if taking oral contraceptives. The physical characteristics (mean ± standard deviation) of the participants are described hereafter. *Protocol 1* (*n *= 6; 3 males, 3 females): age, 22 ± 2 years; height, 1.71 ± 0.06 m; body mass, 69.4 ± 13.7 kg; body surface area, 1.8 ± 0.2 m^2^; body fat percentage, 15 ± 2%; and peak rate of oxygen consumption (VO_2peak_), 39.1 ± 6.5 mLO_2_ kg^−1^ min^−1^. *Protocol 2* (*n* = 6; 6 males): age, 24 ± 5 years; height, 1.74 ± 0.05 m; body mass, 75.7 ± 8.8 kg; body surface area, 1.9 ± 0.1 m^2^; body fat percentage, 15 ± 2%; and VO_2peak_, 49.3 ± 9.3 mLO_2_ kg^−1^ min^−1^. *Protocol 3* (*n* = 6; 5 males, 1 female): age, 23 ± 3 years; height, 1.70 ± 0.10 m; body mass, 72.8 ± 11.8 kg; body surface area, 1.8 ± 0.2 m^2^; body fat percentage, 14 ± 2%; and VO_2peak_, 50.5 ± 8.9 mLO_2_ kg^−1^ min^−1^.

### Experimental procedures

All participants volunteered for one screening session and participated in at least one of three experimental protocols. For a minimum of 48 h prior to each session, participants abstained from prescription and over‐the‐counter medications and vitamin supplements. Furthermore, participants avoided alcohol, caffeine, and heavy exercise for at least 24 h before each session and were instructed not to consume any food 2 h before each session. During the screening session, body height, mass, density, surface area, and fat percentage as well as VO_2peak_ were determined. Body height was measured using a stadiometer (Detecto, model 2391, Webb City, MO), while body mass was measured using a high‐performance digital weighing terminal (model CBU150X, Mettler Toledo Inc., Mississauga, ON, Canada). Body surface area was subsequently calculated from the measurements of body height and mass (Dubois and Dubois 1916). Body fat percentage was calculated (Siri 1956) using body density as determined by hydrostatic weighing. A maximal incremental cycling protocol was performed on a semirecumbent cycle ergometer (Corival, Lode B.V., Groningen, the Netherlands) to assess VO_2peak_. Participants were instructed to maintain a pedaling cadence of 60–90 revolutions per minute at a starting workload of 100 W for males and 80 W for females. Thereafter, the work load was increased by 20 W min^−1^ until volitional fatigue and/or the participants could no longer maintain a pedaling cadence ≥50 revolutions per minute. Throughout the maximal cycling protocol, oxygen uptake was monitored by an automated indirect calorimetry system (MCD Medgraphics Ultima Series, Sun Tech Medical, MN). VO_2peak_ was taken as the highest average rate of oxygen uptake measured over 30 sec.

On the day of the experimental sessions, participants reported to the laboratory well‐hydrated (ensured by consuming 500 mL of water the night prior to and 2 h before the experimental session) and provided a urine sample for the measurement of urine specific gravity before a nude body mass was obtained. Participants then rested in a semirecumbent position in a thermoneutral room (~25°C) while two (Protocols 1 and 2) or four (Protocol 3) microdialysis fibers were inserted into the dermal layer of skin on the dorsal side of the left forearm. All microdialysis fibers were placed in the unanesthetized skin using aseptic technique by first inserting a 25‐gauge needle that exited the skin ~2.5 cm from the insertion point. The microdialysis fiber was then threaded through the lumen of the needle. The needle was then withdrawn, leaving the fiber in place. Each fiber was separated from adjacent fibers by at least 4 cm and secured with surgical tape. The center of each microdialysis fiber was marked on the skin using a fine‐tip permanent marker. Once the procedure was completed, the microdialysis fibers were perfused with lactated Ringer's solution at a rate of 4 *μ*L min^−1^ via a perfusion pump (model 400, CMA, Microdialysis, Solna, Sweden) for ~30–60 min to allow for the resolution of the local hyperemic response.

#### Protocol 1

**Figure 1:**
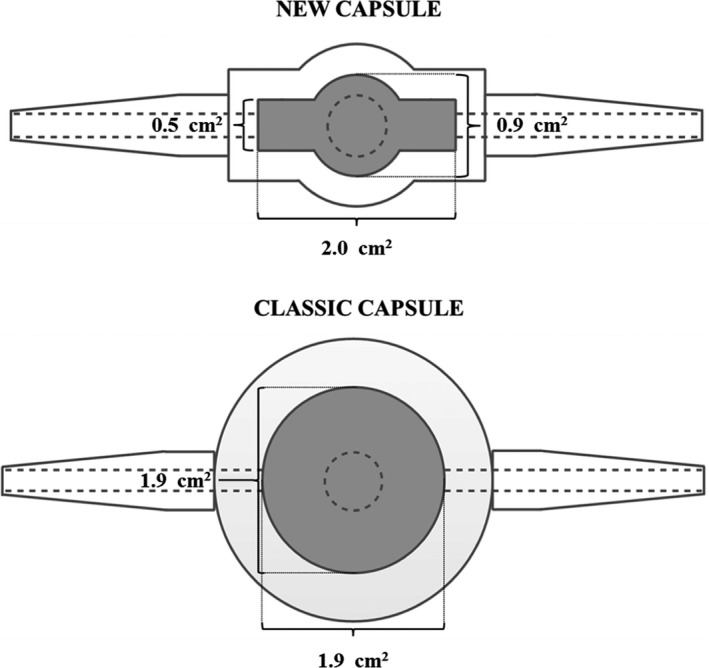
Schematic representation of the new ventilated sweat capsule specifically designed for use with intradermal microdialysis as well as a classic design. The new capsule was designed to encompass the entire skin surface area perfused by the microdialysis fiber, taking into account the fact that microdialysis fibers are longer than they are wide, resulting in an oval shaped perfusion pattern. Note that the new capsules were widened in the center to accommodate a laser Doppler flowmetry probe to allow for the simultaneous measurement of local sweat rate and cutaneous blood flow.

This protocol was designed to evaluate the surface area of forearm skin perfused by the microdialysis fiber using an agonistic approach. Following insertion of the microdialysis fibers (two fibers), a commonly employed, circular ventilated sweat capsule (classic capsule; surface area of 2.8 cm^2^; Fig. 1) was affixed directly over the center of one of the fibers using an adhesive ring and topical skin glue. The two microdialysis probes were then perfused in an incremental manner with five different concentrations of methacholine (molecular weight: 195.69 g mol^−1^; Sigma‐Aldrich, St. Louis, MO), a cholinergic agent known to directly stimulate sweat production (1, 10, 100, 1000, and 2000 mmol L^−1^), at a rate of 4 *μ*L min^−1^. When the forearm sweat rate reached a plateau during each concentration of methacholine, local sweat gland activation was determined at the skin site not instrumented with a ventilated capsule using the modified iodine‐paper technique (Gagnon et al. 2012).

#### Protocol 2

This protocol was designed to evaluate the surface area of skin perfused by the microdialysis fiber during exercise using an inhibitory approach. Following insertion of the microdialysis fibers (two fibers), the forearm was also instrumented with a classic capsule on an area of skin without a microdialysis fiber. Once instrumentation was completed, participants entered a thermal chamber (Can‐Trol Environmental Systems Limited, Markham, ON, Canada) regulated to 35°C and 20% relative humidity and rested quietly on a semirecumbent cycle ergometer (Corival) for a 60‐min habituation period. During this time, the microdialysis fibers were perfused at a rate of 4 *μ*L min^−1^ with either (1) 6 mmol L^−1^ of ouabain (molecular weight: 728.77 g mol^−1^; Sigma‐Aldrich), a Na^+^/K^+^‐ATPase inhibitor; or (2) 58 μmol L^−1^ of atropine (molecular weight: 289.37 g mol^−1^; Sigma‐Aldrich), an anti‐muscarinic agent. These inhibitory agents were chosen due to their marked effect on the sweating response (Sato and Dobson 1969; Sato et al. 1969; Kellogg et al. 1995; Machado‐Moreira et al. 2012). Furthermore, the ability of these agents to inhibit sweat gland activity at these concentrations was confirmed in pilot work using the modified iodine‐paper technique. Following the 60‐min drug perfusion period necessary to establish each blockade, participants performed 30 min of semirecumbent cycling at a fixed rate of metabolic heat production of 400 W to ensure a constant thermal drive for sweating and allow sweat rate to reach steady state. Local sweat gland activation was assessed at each microdialysis site using the modified iodine‐paper technique at the end of the exercise bout.

#### Protocol 3

Based on the results from Protocols 1 and 2, specialized ventilated capsules were designed for use with intradermal microdialysis. A schematic representation of this capsule is presented in Figure 1, along with a schematic of a classic circular capsule design. The size of the new capsule (new capsule; surface area of 1.1 cm^2^; Fig. 1) was chosen to encompass the surface area around each microdialysis probe influenced by drug administration while also (1) providing a surface area large enough to capture an adequate number of sweat glands; and (2) permitting the insertion of a laser Doppler flowmetry probe for the simultaneous measurement of skin blood flow, as is commonly performed. Protocol 3 was designed to verify the ability of the new capsule to accurately measure sweat rate in comparison to a classic capsule. Following insertion of the four microdialysis fibers, participants were transferred to a thermal chamber regulated to 35°C and 20% relative humidity and rested quietly on a semirecumbent cycle ergometer for a 60‐min habituation period. During this time, two of the fibers were perfused at a rate of 4 *μ*L min^−1^ with lactated Ringer's (control), while the other two fibers were perfused with 58 μmol L^−1^ atropine. Atropine was chosen in Protocol 3 given that it resulted in a smaller perfusion area relative to ouabain as well as the methacholine at a dose greater than 1 mmol L^−1^ (see results for Protocols 1 and 2). Two classic capsules were placed over one of each treatment site (i.e., control and atropine), while two new capsules were placed over the remaining sites. All capsules were affixed to the skin using adhesive rings and topical skin glue. Following a 60‐min drug perfusion period, participants performed 30 min of semirecumbent cycling at a fixed rate of metabolic heat production of 400 W, ensuring a constant thermal drive and plateau in the sweating response.

### Measurements

The ventilated capsule technique was employed in all protocols for the purpose of measuring local forearm sweat rate. Compressed anhydrous air was passed through each capsule at a rate of 0.5 L min^−1^ for the classic capsule and 0.2 L min^−1^ for the new capsule. Long vinyl tubes were used to supply the dry gas to and from the ventilated capsules to ensure optimal equilibration with ambient environmental conditions for the experimental sessions. Water content of the effluent air was measured using capacitance hygrometers (Model HMT333, Vaisala, Woburn, WA). Forearm sweat rate was calculated using the difference in water content between the effluent and influent air multiplied by the flow rate and normalized for the skin surface area under the capsule (in mg min^−1^ cm^−2^).

In Protocols 1 and 2, the modified iodine‐paper technique (Gagnon et al. 2012) was used to determine the surface area of skin perfused by the microdialysis fiber at each dose of methacholine (i.e., 1, 10, 100, 1000, and 2000 mmol L^−1^) in Protocol 1 and at the end of the 30‐min exercise bout in Protocol 2. Prior to each measurement, a square piece of cotton paper (3 × 3 cm) that was saturated with iodine was affixed to a flat and hard plastic surface using an adhesive ring. Through a hole in the plastic surface, a blunt guide needle was passed through the iodine paper. This guide needle was then gently placed directly over the center of the microdialysis fiber (marked on the skin by a fine‐tip permanent marker). Thereafter, the iodine paper was firmly pressed onto the skin for a period of ~5 sec. This ensured that the center of each iodine paper corresponded to the center of each microdialysis fiber. All measurements were taken in duplicate at a minimum.

Esophageal temperature was measured continuously in Protocols 2 and 3 using a pediatric thermocouple probe of ~2 mm in diameter (Mon‐a‐therm, Mallinckrodt Medical, St Louis, MO) inserted ~40 cm past the entrance of the nostril. Data were collected using a data acquisition module (model 34970A, Agilent Technologies Canada Inc., Mississauga, ON, Canada) at a sampling rate of 15 sec and simultaneously displayed and recorded in spreadsheet format on a personal computer with LabVIEW software (version 7.0; National Instruments, Austin, TX). Heart rate was recorded using a Polar coded WearLink and transmitter, Polar RS400 interface, and Polar Trainer 5 software (Polar Electro, Kempel, Finland).

In all protocols, pretrial hydration status was verified, to ensure euhydration (urine specific gravity <1.020; American College of Sports Medicine et al., 2007) using a hand‐held refractometer (Reichert TS 400 total solids refractometer, Reichert Inc., Depew, NY).

### Data analyses

In Protocols 1 and 2, the functional perfusion area (i.e., the area of sweat glands measurably activated or inhibited) of the employed agents was determined via the iodine‐paper technique. The height and width of the activated (Protocol 1) or inhibited (Protocol 2) area was assessed by two independent researchers for each sample obtained at each time point (i.e., plateau in local sweat rate for each dose in Protocol 1 and during steady‐state exercise in Protocol 2). Given that the semipermeable membranes of the microdialysis fiber are longer than they are wide, the functional perfusion area was estimated as an oval using the equation:

Surface area=h·w·π

where *h* is the height of the affected skin area in parallel to the direction of the microdialysis fiber and *w* is the width of the affected skin area perpendicular to the direction of the fiber (both in cm).

Local forearm sweat rate was presented as an average of the final 5 min at each dose of methacholine (i.e., 1, 10, 100, 1000, and 2000 mmol L^−1^) in Protocol 1 and the steady‐state sweat rate at 30 min of exercise in Protocols 2 and 3. In Protocols 2 and 3, esophageal temperature and heart rate were calculated as an average of the final 5 min of the 30‐min exercise.

### Statistical analysis

Inter‐reviewer reliability for the surface area of sweat glands that were activated (Protocol 1) or inhibited (Protocol 2) was determined using two‐tailed paired Student's *t* tests as well as Pearson's product–moment correlations (*r*). Furthermore, a one‐way analysis of variance with the factor of dose (five levels: 1, 10, 100, 1000, and 2000 mmol L^−1^ methacholine) was used in Protocol 1 to determine if the dose of the agent influences the surface area of skin perfused. In Protocol 3, a two‐way repeated measures analysis of variance with the factors of treatment site (two levels: control and atropine) and capsule design (two levels: classic [2.8 cm^2^] and new [1.1 cm^2^]) was employed to determine the influence of capsule size on the sweating response with intradermal microdialysis. When a significant main effect was observed, post hoc analysis was carried out using two‐tailed Student's paired *t* tests. For all analyses, the level of significance was set at *P* ≤ 0.05. All values are reported as mean ± standard deviation.

## Results

Based on measurements of urine specific gravity, participants were considered adequately hydrated prior to the start of each protocol (urine specific gravity of 1.013 ± 0.010 in Protocol 1, 1.012 ± 0.011 in Protocol 2, and 1.012 ± 0.011 in Protocol 3).

### Protocol 1

**Figure 2:**
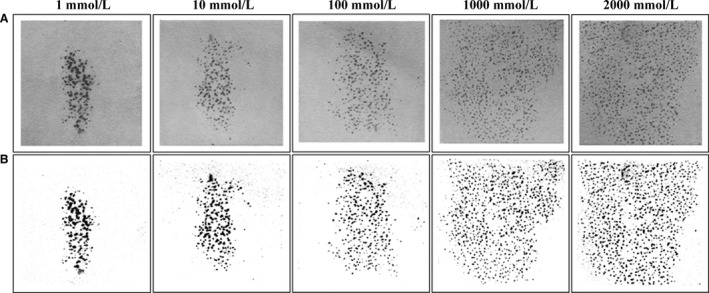
Representative examples of the area of activated sweat glands once plateau was achieved for local sweat rate induced by perfusion of increasing concentrations (i.e., 1, 10, 100, 1000, and 2000 mmol L^−1^) of methacholine. (A) Original scan of the modified iodine paper; (B) edited scan with high contrast to highlight activated sweat glands.

The functional area of skin perfused by each microdialysis fiber, assessed as the area of activated sweat gland activity for Protocol 1, is presented in Table 2 as an overall average as well as for each reviewer individually. In addition, examples of the perfusion area of activated sweat glands induced by methacholine administration are presented in Figure 2. The overall average surface area of activated sweat glands measured at each dose of methacholine was 0.9 ± 0.4 cm^2^ at 1 mmol L^−1^ (range: 0.4–1.4 cm^2^), 1.2 ± 0.6 cm^2^ at 10 mmol L^−1^ (range: 0.3–1.9 cm^2^), 2.3 ± 0.8 cm^2^ at 100 mmol L^−1^ (range: 1.6–3.5 cm^2^), 4.1 ± 1.0 cm^2^ at 1000 mmol L^−1^ (range: 2.7–5.2 cm^2^), and 5.2 ± 1.1 cm^2^ at 2000 mmol L^−1^ (range: 3.9–6.7 cm^2^). Noteworthy, at 1000 and 2000 mmol L^−1^ methacholine, there were several instances wherein the area of activated sweat glands exceeded the height and/or width of the iodine paper (i.e., 3 cm). Therefore, the activated sweat gland area may be larger than the values reported. Furthermore, a main effect of methacholine dose (*P* < 0.01) was observed on the surface area of activated sweat glands such that each incremental dose of methacholine resulted in a progressively greater surface area of activated sweat glands in the forearm skin (all *P* ≤ 0.02). The two‐tailed Student's paired *t* test and correlative analysis revealed a high degree of reliability between reviewers (Table 2). During the administration of methacholine at 1, 10, 100, 1000, and 2000 mmol L^−1^, local sweat rate plateaued at 0.20 ± 0.08, 0.27 ± 0.14, 0.41 ± 0.25, 0.61 ± 0.37, and 0.68 ± 0.31 mg min^−1^ cm^−2^, respectively.

**Table 2:** Measured surface area of skin affected by perfused sudomotor agents via intradermal microdialysis

	Protocol 1					Protocol 2	
	Methacholine (1 mmol L^−1^)	Methacholine (10 mmol L^−1^)	Methacholine (100 mmol L^−1^)	Methacholine (1000 mmol L^−1^)	Methacholine (2000 mmol L^−1^)	Ouabain (6 mmol L^−1^)	Atropine (58 μmol L^−1^)
Reviewer 1 (cm^2^)	0.9 ± 0.4	1.2 ± 0.5	2.3 ± 0.9	4.1 ± 0.8	5.3 ± 1.2	2.1 ± 0.5	1.2 ± 0.3
Reviewer 2 (cm^2^)	0.9 ± 0.4	1.2 ± 0.6	2.3 ± 0.7	4.1 ± 1.2	5.2 ± 1.2	2.1 ± 0.7	1.3 ± 0.3
*P* value	0.82	0.47	0.75	0.93	0.86	0.93	0.87
Correlation (*r*)	0.99	0.98	0.90	0.84	0.94	0.94	0.92
Reviewer mean (cm^2^)	0.9 ± 0.4	1.2 ± 0.5^a^	2.3 ± 0.8^a^	4.1 ± 1.0^a^	5.2 ± 1.1^a^	2.1 ± 0.6	1.3 ± 0.4

Values presented as mean ± standard deviation. In Protocol 1, methacholine was administered in a dose‐dependent manner and the area of activated sweat glands was measured using the modified iodine technique. In Protocol 2, ouabain and atropine were administered during 30 min of exercise at a fixed rate of metabolic heat production of 400 W and the area of inhibited sweat glands was measured using the modified iodine‐paper technique. *P* value represents the output of two‐tailed Student's paired *t* tests between values for reviewer 1 and reviewer 2. Correlation (*r*) represents Pearson's product–moment correlations between values for reviewer 1 and reviewer 2. Reviewer mean values are an average of the values measured by reviewer 1 and reviewer 2.

a Significant difference versus preceding dose in Protocol 1. Note: In Protocol 1 there were several instances of activated sweat gland areas exceeding the height and/or width of the iodine paper. Therefore, activated sweat gland area may be larger than values reported.

### Protocol 2

**Figure 3:**
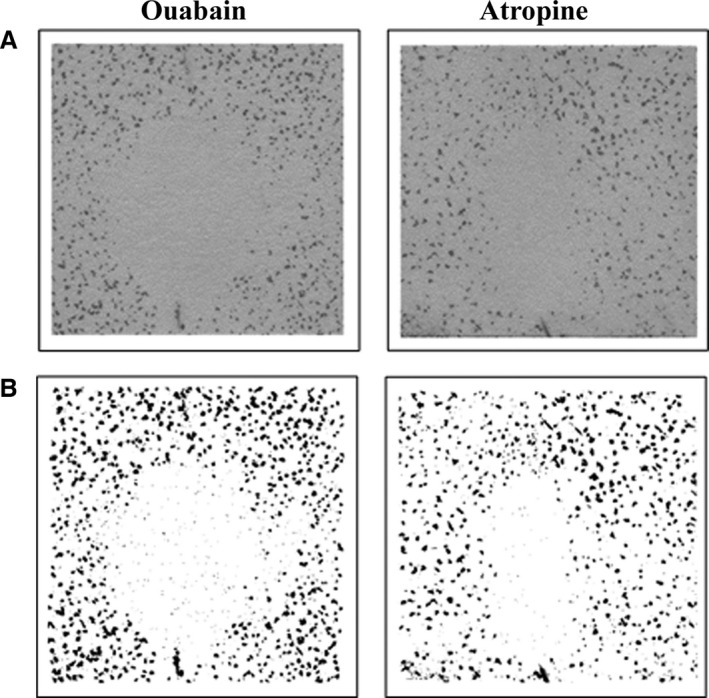
Representative examples of the area of inhibited sweat gland activity induced by administration of 6 mmol L^−1^ ouabain and 58 μmol L^−1^ atropine during steady‐state exercise at a fixed rate of metabolic heat production of 400 W. (A) Original scan of the modified iodine paper; (B) edited scan with high contrast to highlight activated sweat glands.

The surface area of affected skin perfused by each microdialysis fiber for Protocol 2, assessed as the area of inhibited sweat gland activity, is presented in Table 2. Illustrated examples of inhibited sweat gland activity induced by ouabain and atropine are also presented in Figure 3. At the ouabain‐treated skin site, the overall average surface area of inhibition was 2.1 ± 0.6 cm^2^, with a range of 1.2–2.7 cm^2^. At the atropine skin site, the overall average surface area of inhibition was 1.3 ± 0.3 cm^2^ with a range of 1.0–1.7 cm^2^. Statistical analysis revealed a high degree of reliability between reviewers (Table 2). During exercise, esophageal temperature and local sweat rate achieved steady‐state values of 37.56 ± 0.44°C and 0.82 ± 0.16 mg min^−1^ cm^−2^, respectively. During this time, heart rate was 117 ± 14 bpm.

### Protocol 3

**Figure 4:**
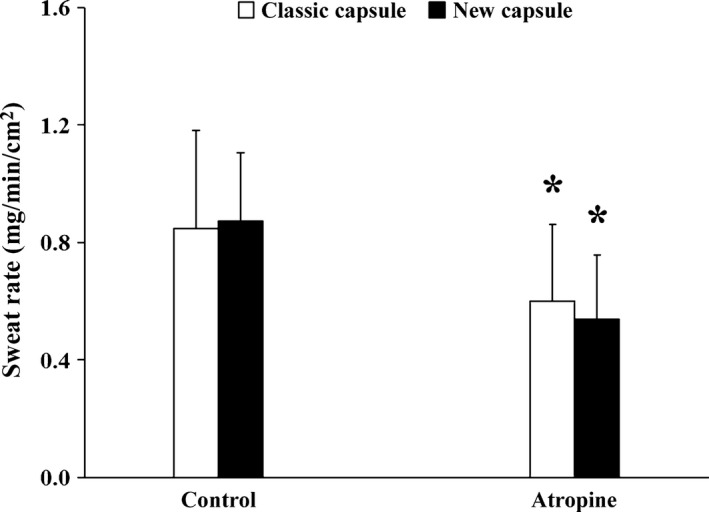
Local sweat rate during exercise at a fixed rate of metabolic heat production of 400 W after the sweating response had reached steady state. Sweat rate was measured at skin sites continuously perfused via intradermal microdialysis with lactated Ringer's (two sites), to act as a control, or atropine (two sites), an anti‐muscarinic agent with a classic capsule (white bars) as well as a new capsule (black bars). Values are mean ± standard deviation. Data are presented as a 5‐min average of data collected after sweat rate had reached steady state (exercise time of ~30 min). *Control significantly different from atropine; *P* ≤ 0.05.

A comparison of the local sweating response between the classic and newly designed ventilated capsules at the control and atropine‐treated skin sites are illustrated in Figure 4. A main effect of treatment site was observed (*P* < 0.01) such that local sweat rate at the atropine site was lower than the control site when measured with both the classic (control: 0.85 ± 0.33 mg min^−1^ cm^−2^ vs. atropine: 0.60 ± 0.26 mg min^−1^ cm^−2^; *P* = 0.05) and new (control: 0.87 ± 0.23 mg min^−1^ cm^−2^ vs. atropine: 0.54 ± 0.22 mg min^−1^ cm^−2^; *P* < 0.01) ventilated capsules. Importantly, neither an interaction of ventilated capsule design and treatment site (*P* = 0.23) nor a main effect of capsule design (*P* = 0.78) were observed. During exercise, esophageal temperature reached steady‐state values of 37.70 ± 0.50°C, while heart rate was 113 ± 30 bpm.

## Discussion

The current study sought to evaluate the surface area of skin perfused via intradermal microdialysis by assessing sweat gland activation and inhibition during methacholine administration (Protocol 1) and exercise (Protocol 2), respectively. We demonstrate marked variability in the functional skin perfusion area (0.9 ± 0.4 cm^2^ to 5.2 ± 1.1 cm^2^), which was strongly dose dependent. From these findings, a new ventilated capsule was designed specifically for use with intradermal microdialysis. This capsule (Fig. 1) was created longer than it is wide to account for the oval shaped perfusion area created by the microdialysis fiber (Figs. 2 and 3). Contrary to our hypothesis, comparisons of the local sweating response induced by atropine perfusion between the new and classic capsule designs (Protocol 3) yielded similar responses, suggesting that capsule size is not a factor when determining the mechanisms of local sweating using microdialysis.

With the recent utilization of the ventilated capsule for assessing local sweat rate in combination with intradermal microdialysis, much work has been done to characterize factors that influence sweating at the level of the end organ – the sweat gland (Inoue et al. 1999; Kondo et al. 2001; Gagnon and Kenny 2011; Machado‐Moreira et al. 2012; Fujii et al. 2014, 2015a,b,d; McGinn et al. 2014; Stapleton et al. 2014a). However, capsule design has not been standardized between laboratories, resulting in a variety of different capsule sizes (Table 1) with surface areas ranging from ~0.5 cm^2^ (Lorenzo and Minson 2010) to ~4.5 cm^2^ (Smith et al. 2013). The surface area of the capsule employed may therefore impact the mechanistic insight gained from this technique if the surface area of the capsule was larger than that of the area of skin perfused via microdialysis, resulting in the capture of sweat glands not influenced by the delivered sudomotor agent. However, until the current study, information on the area of skin perfused by the microdialysis fiber was not available. To evaluate the functional area skin perfused by sudomotor agents delivered via microdialysis, we measured the skin area of sweat gland activation using an agonistic approach wherein methacholine (a cholinergic agonist) was administered in a dose‐dependent manner (Protocol 1). Additionally, we employed an antagonistic approach wherein ouabain and atropine, two agents shown to markedly attenuate the sweating response (Sato and Dobson 1969; Sato et al. 1969; Kellogg et al. 1995; Machado‐Moreira et al. 2012), were administered during exercise and the surface area of inhibited sweat gland activity was evaluated (Protocol 2). Our results show marked variability in the functional surface area of skin perfused by the microdialysis fibers, with values ranging from ~0.9 cm^2^ to ~5.2 cm^2^. (Note: in Protocol 1 there were several instances wherein the area of methacholine‐activated sweat gland exceeded the height and/or width of the iodine paper. Therefore, the actual surface area of activated sweat glands may be larger than values reported.)

Importantly, our results demonstrate that the size of the functional perfusion area was largely dose dependent. This can be clearly observed in our results for Protocol 1, wherein the area of activated sweat glands induced by each dose of methacholine (1, 10, 100, 1000, and 2000 mmol L^−1^) was larger than each preceding dose (i.e., the area was lowest at 1 mmol L^−1^ and highest at 2000 mmol L^−1^ with a progressive increase at each concentration). The influence of agent concentration on the perfusion area is also indirectly supported by the results of Protocol 2, such that the inhibited sweat gland area induced by ouabain (6 mmol L^−1^) was ~0.8 cm^2^ greater than that of atropine (58 μmol L^−1^). However, concentration alone does not explain the functional perfusion area of each agent. Specifically, atropine, which was administered at a concentration of 58 μmol L^−1^ during exercise in Protocol 2, resulted in an area of inhibited sweat glands that was greater than and similar to the area of activated sweat glands induced by 1 and 10 mmol L^−1^ of methacholine, respectively, during passive rest in Protocol 1. In accordance with Fick's law of diffusion, increases in skin temperature as well as differences in interstitial fluid pressures associated with the performance of exercise in a hot environment may have affected the spread of the employed agent from the microdialysis fiber and thereby the functional perfusion area observed. However, given that different agents were employed between Protocols 1 (passive rest) and 2 (exercise), we are not able to directly comment on the influence of these factors.

The current findings have several important implications for the use of intradermal microdialysis in combination with the ventilated capsule technique. Primarily, if pharmacological agents are employed in low concentration and/or the distance of perfusion from the microdialysis fiber of the utilized agent is small, a specialized capsule is ideal in order to completely encompass the skin perfusion area without capturing sweat glands that are not influenced by the agent. However, when larger concentrations of a given agent are employed, a larger capsule may be beneficial given that a larger number of sweat glands would be captured thereby reducing the influence of any heterogeneity between skin sites in the distribution of sweat glands (Taylor and Machado‐Moreira 2013) on the observed response. With this in mind, the data gathered from Protocols 1 and 2 were used to develop a new ventilated capsule specifically for use with intradermal microdialysis. The schematic of this capsule can be viewed in Figure 1. This capsule was designed to take into account the fact that the microdialysis fiber is longer than it is wide, which results in an oval shaped perfusion pattern (such as those depicted in Figs. 2 and 3) while also capturing an adequate number of sweat glands. The purpose of Protocol 3 was to evaluate the local sweat rates measured with this new capsule design during exercise and to compare them to a classic larger circular capsule. Despite the different designs, both capsules resulted in similar sweat rates (after accounting for skin surface area) during exercise at the control site which was paralleled by a similar attenuation in sweat rate during the perfusion of atropine. These findings show that the classic capsule design, which has been widely used in the past, can accurately characterize the sweating response during exercise when employed with microdialysis relative to the new capsule design.

Given that the perfusion area of atropine during exercise (1.2 ± 0.3 cm^2^) was reportedly smaller than the surface area of the classic capsule, the reason underlying the similar responses between capsule types is unknown. These findings may potentially be explained by the fact that the modified iodine‐paper technique allowed us to determine only the area of sweat glands completely inhibited by atropine (i.e., the functional perfusion area). However, it is likely that the concentration and thereby the influence of atropine was progressively reduced from the mircodialysis fiber to the periphery of the perfusion area. Therefore, it is possible that the sweat output of the activated sweat glands observed just outside of the area of inhibition was markedly attenuated but not completely abolished. Regardless, our results suggest that use of the classic capsule does not impact the mechanistic implications to the sweating response. Indeed, a study employing large circular capsules recently reported minute (~0.05 mg min^−1^ cm^−2^) differences in local forearm sweat rate at a skin site perfused with angiotensin II in comparison to a control site (Fujii et al. 2015d). Altogether, it is suggested that the new capsule design be used to better encompass the surface area of skin perfused by the microdialysis fiber, especially if the concentration of the agents employed is small and/or they exert a less pronounced effect on the sweating response (e.g., angiotensin II [Fujii et al. 2015d]). However, the use of larger classic capsules is still valid and likely results in decreased between‐site variability given that they capture a larger number of sweat glands, which would reduce the influence of heterogeneity in sweat gland distribution on the measured response (Taylor and Machado‐Moreira 2013).

### Considerations

An important consideration of the current study is that the area of skin perfused by each microdialysis fiber could not be directly determined. Rather, we used a practical approach whereby the functional perfusion area was determined based on the area of skin wherein the sweating response was affected (i.e., activated or inhibited). However, it is possible that agents employed migrated further from the capsule than reported but were not able to completely influence the sweating response at these sites. Furthermore, because we employed the modified iodine‐paper technique, which gives information regarding the number of activated sweat glands, the agents used in the study were confined to those that activated or inhibited the sweating response and not agents that have been shown to modulate but not directly stimulate sweating such as nitric oxide (Fujii et al. 2015e). Regardless, our findings provide important information regarding the perfusion characteristics of agents administered to the skin via microdialysis. Along with the new capsule design, this study provides direction for studies concerned with precisely evaluating the mechanisms underpinning the sweating response. However, future work should be conducted to determine how factors including age and sex, which have been shown to modulate the sweating response (Gagnon and Kenny 2011, 2012; Larose et al. 2013), may influence the diffusion of employed agents in the skin.

## Conclusion

The findings of the current study provide novel methodological information regarding the surface area of skin influenced by sudomotor agents delivered via microdialysis. Specifically, it was determined that marked variability in the diffusion of sudomotor agents in the skin exists that was largely dose dependent, such that the area of skin perfused was 0.9 ± 0.4 cm^2^ to 5.2 ± 1.1 cm^2^. Furthermore, a new specialized ventilated capsule is presented, specifically designed to optimally capture the area of skin perfused by the microdialysis fiber; albeit, no differences in the attenuation in local forearm sweating in response to atropine infusion were observed in comparison to a classic capsule. Altogether, we recommend the use of the newly designed, specialized sweat capsule in situations in which small doses of the employed agent are required and/or the perfusion area of the selected agent is small. However, in most cases it does not appear that use of a classic capsule will alter the mechanistic implications to the sweating response gleaned via intradermal microdialysis.

## Conflict of Interest

None declared.
